# Practical Challenges of Current Video Rate OCT Elastography: Accounting for Dynamic and Static Tissue Properties

**DOI:** 10.4172/2469-410X.1000112

**Published:** 2014-12-12

**Authors:** Mark E Brezinski

**Affiliations:** 1 Center for Optics and Modern Physics, Brigham and Women’s Hospital, 75 Francis Street, Boston, M.A. 02115, USA; 2 Harvard Medical School, 25 Shattuck Street, Boston, M.A. 02115, USA; 3 Department of Electrical Engineering, Massachusetts Institute of Technology, 77 Massachusetts Avenue, Cambridge, M.A. 02139, USA

**Keywords:** Optical Coherence Tomography, Elastography, Thin-capped fibroatheromas

## Abstract

Optical coherence tomography (OCT) elastography (OCTE) has the potential to be an important diagnostic tool for pathologies including coronary artery disease, osteoarthritis, malignancies, and even dental caries. Many groups have performed OCTE, including our own, using a wide range of approaches. However, we will demonstrate current OCTE approaches are not scalable to real-time, in vivo imaging. As will be discussed, among the most important reasons is current designs focus on the system and not the target. Specifically, tissue dynamic responses are not accounted, with examples being the tissue strain response time, preload variability, and conditioning variability. Tissue dynamic responses, and to a lesser degree static tissue properties, prevent accurate video rate modulus assessments for current embodiments. Accounting for them is the focus of this paper. A top-down approach will be presented to overcome these challenges to real time in vivo tissue characterization. Discussed first is an example clinical scenario where OTCE would be of substantial relevance, the prevention of acute myocardial infarction or heart attacks. Then the principles behind OCTE are examined. Next, constrains on in vivo application of current OCTE are evaluated, focusing on dynamic tissue responses. An example is the tissue strain response, where it takes about 20 msec after a stress is applied to reach plateau. This response delay is not an issue at slow acquisition rates, as most current OCTE approaches are preformed, but it is for video rate OCTE. Since at video rate each frame is only 30 msec, for essentially all current approaches this means the strain for a given stress is changing constantly during the B-scan. Therefore the modulus can’t be accurately assessed. This serious issue is an even greater problem for pulsed techniques as it means the strain/modulus for a given stress (at a location) is unpredictably changing over a B-scan. The paper concludes by introducing a novel video rate approach to overcome these challenges.

## Introduction

Optical Coherence Tomography Elastography (OCTE) has the potential to be an important diagnostic tool for pathologies including coronary artery disease, osteoarthritis, malignancies, and even dental caries. Many groups, including our own, have performed OCTE using a wide range of approaches. However, current OCTE approaches are not scalable to video rate modulus assessments. While considerable effort has gone into techniques for applying stress and measuring tissue changes, there are substantial challenges that are overlooked largely because they play little role at slow acquisition rates. The most significant of these are the tissue responses (and to a lesser degree static tissue properties), such as tissue strain response times, preload variability, and conditioning variability. This paper examines these limitations and explores overcoming them through a top down approach. First, an example clinical scenario is discussed where OCTE could play an important role in patient morbidity and mortality. This is in the prevention of myocardial infarction or heart attacks. Second, the principles behind OCTE will be examined. Third, the limitations tissue properties (dynamic and static) place on current OCTE, for in vivo video rate assessments, will be discussed. These are a minor issue at slow acquisition rates but prevent current approaches from being scaled up to real time. Finally, we introduce one approach to overcoming these limitations. For this approach, one component is that stress is applied with constant frequency, amplitude-modulated ultrasound. The amplitude is changed between two values on every first and third frame so that tissue strain plateaus during measurement on every second and fourth frame. While this general approach overcomes the obstacles, the approach is not limited to a specific embodiment as will be seen.

### Example relevant clinical scenario

Acute coronary syndromes (ACSs), myocardial infarction and unstable angina, are the leading cause of death in the industrialized world, representing over 25% of all mortalities in the US alone [1]. The importance of early aggressive intervention in the highest risk groups can’t be overemphasized. In addition to intense global risk factor reduction, such as cholesterol management, selective catheter-based intervention on high-risk plaques would be a highly significant advance in reducing mortality. But direct intervention on these plaques is generally not possible because of the limited ability to identify and risk-stratify thin-capped fibroatheromas (TCFAs), the predominate plaque that leads to ACSs [2]. Plaque strain analysis and autopsy data has demonstrated TCFAs are characterized by intimal caps less than 100 μm thick and have soft necrotic cores (high strain), making them susceptible to rupture [2–14]. When these plaques rupture, they release thrombogenic necrotic material into the blood, a clot forms, and the vessel occludes in about 20% of the cases [2–19]. We have recently demonstrated the TCFAs in proximity to long longitudinal necrotic cores or shafts may represent this 20% at greatest risk of progression to ACSs1 [20]. *The role of OCTE in this clinical scenario will be both to identify those thin-walled plaques that have necrotic cores as well subclassifying them to the highest strain*.

In the early 90s we introduced optical coherence tomography (OCT) for assessing high-risk plaque, which is particularly effective for identifying thin intimal caps on plaques [21]. OCT, which is reviewed elsewhere, is a micron scale imaging modality based on the back reflection of infrared light [22–27]. Human intravascular imaging was performed in the first decade of the millennium and it was FDA approved in 2010 for coronary imaging [28–31]. Time domain OCT (TD-OCT) was the dominant OCT embodiment for over a decade, but the higher acquisition rate of swept source OCT (SS-OCT), which frequency sweeps the source and reconstructs backscattering indirectly, is now the standard for intravascular imaging. While OCT provides improved delineation of TCFAs relative to other technologies, both limited use of certain capabilities as well as misconceptions of the efficacy of others has restricted its utility [30–36]. One of these capabilities is OCTE. We have recently reviewed OCT limitations/misconceptions in several publications including papers in *Nature Cardiology Reviews* as well as *Circulation* and *JACC* (in addition to several open access papers that are more extensive) [27–33,35]. These serious misconceptions include overestimating its efficacy for identifying necrotic cores, needed for recognizing TCFAs.

Necrotic core identification is critical for selective plaque intervention because TCFAs lead to most ACSs. The necrotic cores are both thrombogenic and soft (high strain), the latter predisposing to rupture. Primarily fueled by one study (Yabushita et. al), a commonly held but incorrect belief (for over a decade) is that current OCT image interpretation reliably identifies necrotic cores [23,25,36]. The authors claimed ‘diffuse borders’ between the intima and core identify lipid plaque, which we have demonstrated is highly unlikely to be correct. Further supporting this is incorrect is that the study treated necrotic core and lipid collections as identical, which is incorrect and a distraction to the field. The conclusion of the study was that the ‘diffuse border’ criteria only had a 70% predictive power for lipid plaque (with no insight into necrotic plaque*)*. But this criteria is being used in clinical trials claiming incorrectly to identify necrotic cores with no experimental data to support the conclusion. The problems should have been obvious from the author’s own observations “False-positive OCT diagnoses of lipid-rich plaques often contained histological evidence of small amounts of lipid present within a predominantly fibrous plaque.” We have produced theory and data, in numerous publications, why the diffuse border criteria is incorrect and an alternate assessment approach is needed [21,27–33] Two additional smaller studies had even less promising results using the diffuse border criteria; one of these studies reported only a 45% sensitivity and 83% specificity for identifying lipid-filled plaques [24–26]. Our conclusions, that current OCT does not identify necrotic cores, have been supported recently by the *2012 Consensus Standards for Acquisition, Measurement, and Reporting of Intravascular Optical Coherence Tomography Studies* [36]. The problem is these important conclusions were not stated prominently in the paper by the panel. In the text the panel wrote “At present, there are no definitive published studies directly comparing OCT lipid pool–containing plaques with necrotic core by histology, and as a result, the Evidence Level was Determined to be Low for OCT delineation of necrotic core.” In other words, saying a lesion has a necrotic core by OCT imaging is not based on any data in the literature. All our concerns with the Yabushita et al. paper can’t be addressed in this limited space here but we have extensively reviewed this topic elsewhere [23,27–30]. Since current OCT approaches do not identify necrotic cores, OCTE represents a promising alternate approach for identifying necrotic core.

### Principles of Optical Coherence Tomography Elastography (OCTE)

OCTE has the potential of addressing many clinical issues, particularly identifying necrotic cores and especially those with high strain. Elastography, as discussed below, measures tissue displacement (strain) within images, under a controlled stress, to assess mechanical properties (modulus) [37–40]. With elastography, when an applied axial force is known and the strain is measured, then the Young’s modulus can be estimated (essentially stress divided by strain) [41–43]. This assumes tissue is relatively uniform, incompressible, and an isotropic linear elastic material, which we will demonstrate is valid for the approach proposed. Our group and many others have developed OCTE techniques at slow data acquisition rates [43–82]. But only several groups, including ours, have validated these techniques against standards. Similarly only a small number of groups have performed OCTE at video rate and we are unaware of a video rate study validated against standards. The amount of OCTE work is extensive so will not be listed here (several examples are outlined in the discussion). Instead, in the next section, we discuss the different components of OCTE and this encompasses all the different OCTE techniques.

### OCTE components

OCTE approaches, and elastography in general, can be viewed as consisting of five components (many publications simply divide them just into static versus dynamic stress techniques). Understanding these components is critical to developing OCTE for in vivo vascular assessments of TCFA. The five components of OCTE are the stress application, modality used to measure tissue changes in response to stress, approach to analyze measured tissue changes, static tissue properties, and dynamic tissue properties in response to the applied stress. All OCTE techniques are a combination of these.

It is the last two that are the focus of this paper because they are rarely recognized issues but they limit in vivo utility of most (if not all) current OCTE techniques. They dictate how the stress is transformed into strain. With regard to system design, the fourth influences the intensity and area of the applied stress. The fifth limits when the strain can be measured. Not taking into account these last two has limited real-time OCTE.

#### The components are

##### 1. Methods for applying intravascular stress

The various approaches of applying stress to a plaque include: A. direct contact pressure, generally from an expandable intravascular balloon, B. fluid pressure from saline flushes or timing to blood pressure changes, and C. ultrasound (including pulsed such as acoustical radiation force). The first two do not produce reliable pressures and are not discussed here. [21,58]

##### 2. Technology for measuring strain

For intravascular elastography, the modulus/strain is generally measured either with IVUS or OCT. *A. IVUS:* IVUS has superior penetration to OCT. Its disadvantages include the need to also use OCT for assessing other structures (ex: intimal cap width) and the large area over which elastography data is averaged. In addition, IVUS produces pressures (from the ultrasound) in excess of 100 kPa (>250 mm Hg much higher than TCFA moduli), the influence of which is discussed under component five (causing high irregular pre-loads and conditioning [55] B. *OCT*: OCT offers many advantages for elastography assessments of plaque. First, it allows, along with elastography measurements, simultaneous assessment of plaque microstructure less than 20 μm. Second, unlike ultrasound, light produces no pressure so there is no preloading or conditioning. Third, since ultrasound is not used for assessment, ultrasound can be used for controlled applied stress (pulsed or CW). A limitation of OCT is penetration. But while it is an issue when assessing core depth, it is not a major issue for OCTE. This is because we are primarily interested in the mechanical properties of the core near the cap center, so penetration is not an issue.

##### 3. Three approaches for analyzing the modulus

**Figure 1:**
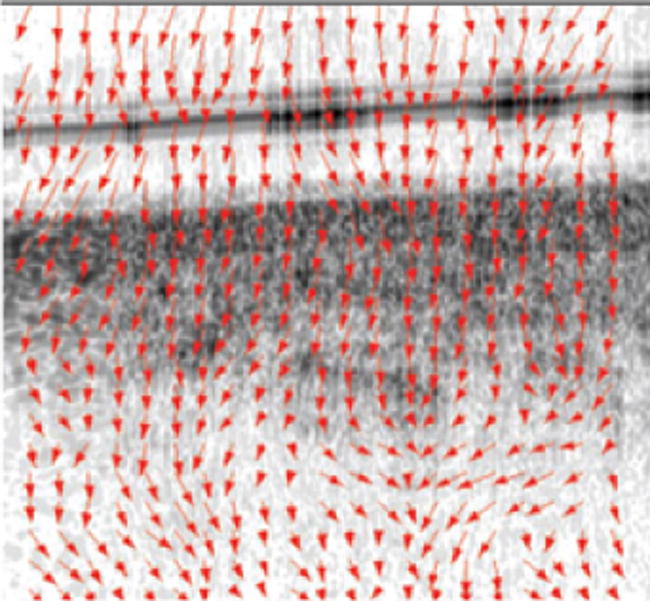
Example Elastogram. The length of the arrow is the amount of displacement.

The three most common approaches that attempt to measure the modulus through tissue movement (under controlled stress) are speckle displacement, phase changes, and dynamic approaches. While elegant, the phase-based approaches with their high resolutions are not optimal in vivo as they are too sensitive to noise and tissue movement. The dynamic approaches attempt to model a complex varying strain. This is challenging to achieve, as it involves many assumptions on tissue properties that may be inaccurate, but it also has similar sensitivity issues to the phase approaches [48–57]. With sufficient resolution for assessing plaque and less sources of error, available data strongly suggests speckle-based approaches are optimal for in vivo application. Speckle is described elsewhere, but it basically is subcoherence length interference between particles giving pixel-to-pixel variations. Speckle shifts with stress and can therefore identify small displacements [58–59]. The most common speckle tracking elastography technique is the cross correlation function, which we have used previously and is discussed elsewhere [42,43,46,47]. Figure 1 shows displacement vectors within an atherosclerotic plaque, where the length of the arrow demonstrates amount of displacement. Among the merits of the cross correlation function are decreased noise, having been extensively tested, and minimal vessel movement artifacts (relative to phase and dynamic approaches). A significant limitation is that the adaptive window needs to be carefully designed for optimal performance (if too small, it is insensitive and, if too large, it will average out values). We have already generated data in arteries supporting a 21×21 pixel as the optimal correlation window for plaque [42–43]. Loss of the speckle pattern from too large displacements must be prevented and so de correlation measurements are important in determining the applied stress.

These first three components are the most focused on in OCTE research but the next two, the fourth and fifth, are both the most overlooked and the most critical to take into account for video rate OCTE. These components, and the challenges they introduce, are one of the most critical aspects of the paper.

##### 4. Static tissue properties

Static tissue properties include the size, shape, amount of compression, and composition of the necrotic core. For measurements to be accurate, these static tissue properties need to be accounted for. Specifically they determine the appropriate area to apply stress (location and size) and what pressures can be applied (which for TCFAs is relatively low, 5–25 kPa). These are important in maintaining linear properties as numerous studies looking at plaque elastic properties demonstrate measurements are accurate (i.e. hold within 2% of the actual modulus and are linear) when: 1) the lipid core modulus is higher than the surrounding tissue, 2) the core is larger laterally than axially, 3) the compressions are only over the core (and not adjacent intima), 4) compressions are less than 5%, and 5) applied pressures are near the tissue modulus [83–89]. The first two hold for TCFAs microstructure and the other three are *dictated by the sampling area and optimal applied pressures. We will see that the beam size needs to be smaller than the TCFA diameter (less than 2 mm) and the applied pressures much less than 100 kPa. These are essentially universally overlooked in OCTE design, but they need to be critical to accurate modulus measurements.* Numerous studies have demonstrated that, when these conditions are met for the small plaques we are interested in, heterogeneous lipid cores behave as if they are relatively homogeneous (i.e. regional assessments are representative [83–89]. This would not hold for IVUS elastography because of its low resolutions, which would result in areas outside the core being sampled and high irregular pressures associated with IVUS (>100 kPa).

##### 5. Dynamic tissue responses

**Figure 2:**
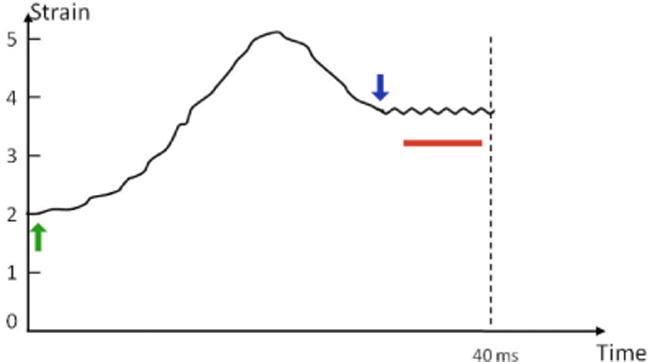
The tissue strain response to a cw stress. The green arrow is the applied stress, the blue arrow when plateau is reached, and the red line conditioning/oscillations. Pleateau takes a finite time to reach.

The dynamic tissue responses may be the most critical challenge to video rate in vivo OCTE, but they are the least considered factors in system design. But these dynamic responses are a main focus of this paper. Of particular importance, which can alter the measured modulus unpredictably, are the pre-load, conditioning, and finite tissue response times. Because of these, at high acquisition rates, stress generally does not under most circumstances translate to a highly reproducible modulus. Therefore, stress needs to be applied with an understanding of the dynamic tissue responses to improve accuracy. These factors are not a major issue at low data acquisition rates, but as will be seen they become extremely important at high data acquisition rates and with pulsatile stresses as dynamic tissue responses will lead to inaccurate modulus measurements (when not compensated for). In other words, the measured modulus is a function of the pre-load, conditioning, and tissue response time. We provide an approach that accounts for and controls these factors. Figure 2 looks at the response to a CW stress. *First*, the pre-load (green arrow in Figure 2), the lowest baseline force on the tissue, can substantially alter the modulus. It therefore should be accounted for and kept constant. Generally, the preload on the vessel wall (in vivo) is the diastolic blood pressure (approximately 10 kPa = 80 mm Hg) but when IVUS is used, the ultrasound becomes the preload because of the higher magnitude (greater than 100 kPa) [55]. We will demonstrate one way of controlling it by using the lower of two ultrasound CW amplitudes as the pre-load. It should be noted that it is challenging to keep the preload constant with a pulsed source, which is why we use the CW approach outlined. *Second*, conditioning (red line in Figure 2), or rhythmically altering stress on the tissue, can decrease the modulus so it needs to be controlled. We will be applying a constant carrier frequency, to the CW ultrasound, that provides constant conditioning. *Third*, although rarely appreciated but likely the most critical limitation, there is a finite but substantial response time for the strain to plateau (blue arrow) after the stress has been applied. In general the tissue strain response reaches near plateau at 20 msec [90–92]. For slow acquisition rates, which are how the vast majority of OCTE experiments have been performed, the tissue has plateaued after CW stimulation during most of the B-scan. But when considering B-scans at video rate (approximately 30 msec/frame), the problem becomes clear. This means the strain for a given CW stress is constantly changing during one B-scan. Therefore, sophisticated modeling (assuming it could be modeled) would be required even if the pre-load and conditioning are unaffected. Our approach avoids the need for this complex modeling. For a pulsed stress the situation is worse, where modeling of pulses to date does not account for this. Using the plateau phase (red line), we have developed a strategy to keep the strain, preload, and conditioning relatively constant during OCTE measurements.

## Overcoming the Challenges of Static and Dynamic Tissue Responses for Video Rate OCTE

### Proposed embodiment for video rate OCTE

**Figure 3:**
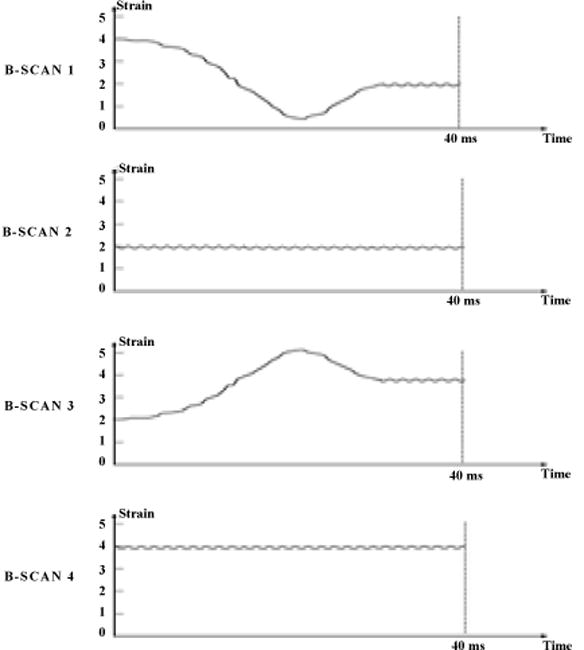
Ultrasound amplitude is changed in the frames 1 and 3. OCTE measurement made in frames 2 and 4.

OCTE at video rate faces challenges posed by static and dynamic tissue responses. Using a top down methodology accounting for these factors, we propose a *general OCTE approach* at video rate that can be used with a variety of physical embodiments (with one described here). Here, with respect to dynamic properties, CW ultrasound at two amplitudes (amplitudes discussed below) is used as the applied stresses (but the approach is not restricted to ultrasound). The lower ultrasound value is the preload and the ultrasound carrier frequency is used for conditioning. The ultrasound are changed between every other frame (1^st^ and 3^rd^ frames) while measurements are made in the other two frames (2^nd^ and 4^th^). This is seen in Figure 3. By transitioning CW stress amplitudes in between measurement frames, rather than trying to measure the modulus while it is fluctuating, it accounts for the dynamic tissue properties (strain response time, pre-load and conditioning) without the need for complex modeling (strain has plateaued).

To address the problems associated with static tissue properties, we propose a focused beam size of 2 mm on the plaque. This beam size will not cover the plaque edges (TCFAs of interest exceed 2 mm in diameter). In our previous examination of fibroatheromas and fibrous plaques for OCTE, the area assessed was 50×50 pixels, with a 21×21 kernel window, and 500 μm by 50 μm imaging area. The same will be used here. The applied ultrasound pressure is in the range of TCFA moduli (<75 kPa) which is much lower than that used in previous OCTE. How the ultrasound amplitude will be identified is discussed below.

**Figure 4:**
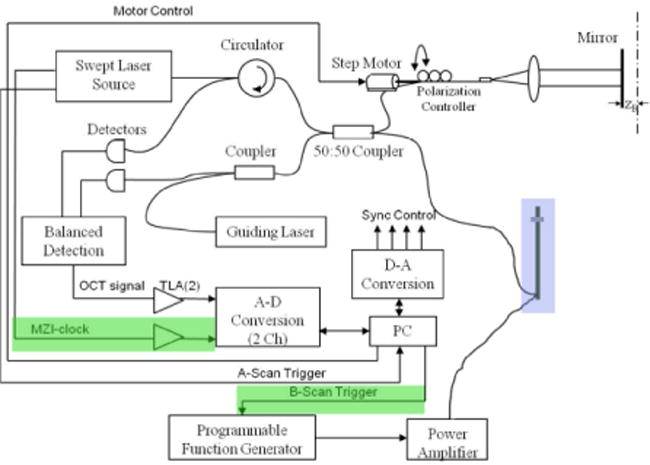
Schematic of SS-OCT/Ultrasound System. The green shows the synchronization between the source sweep and the ultrasound changes in Figure 3.

A schematic of the proposed system design is shown in Figure 4 [48–50,60–77]. The SS-OCT imaging system is a Light lab C7-XR FD-OCT Intravascular Imaging System (St. Jude Medical). A modified C7 Dragonfly Imaging Catheter will be used (St. Jude Medical). The transducer on the catheter is discussed below. This transducer is driven with an amplitude-modulated signal from an arbitrary waveform generator (Tabor Electronics 8026), amplified by a ENIA-500 power amplifier. The available amplitude range needs to be between 5 kPa to 250 kPa to cover the range potentially needed, making the system versatile. It is anticipated the lower amplitude value will be 10 kPa (diastolic pressure) and, based on current knowledge of lipid plaque moduli, have an upper value between 50–75 kPa. Synchronization is needed between ultrasound amplitude changes and SS-OCT sweeps. Therefore, since the software in SS-OCT generates a trigger signal, this is achieved by outputting through the TTL port to the function generator (Figure 4) [93,94]. The amplitude changes begin 5 ms into the 1^st^ and 3^rd^ B-scans (Figure 3). As previously stated, the carrier frequency serves as the means to control conditioning. Based on work by our group and others, speckle modulations by the carrier decreases with increasing frequency (negligible at 1 kHz) until it reaches approximately 10 MHz (and greater) where particles begin Doppler shifting the OCT signal. The optimal frequency will be near 1 MHz, which needs to be confirmed by performing self-correlations of A-scans (for the highest and lowest strain phantoms). Here the correlation function in the optimal frequency range should not be significantly different from zero.

Our preliminary studies on the speed of this cross correlation approach are shown in Table 1 and suggest there is no need to change to either a generic algorithm, phase analysis, or other speckle approaches. As can also be seen, rapid analysis is achieved even with a relatively slow computer (5 year old Apple, 2.26 GHz). As stated, a kernel window size of 21 × 21 based on previous work is used. We are interested primarily in the average displacement for the area of interest. Absence of decoherence needs to be confirmed.

### This approach deviates from the status quo by at least five criteria

#### 1. Use of applied stresses near that of TCFAs

Based on prior work, plaques high in lipid have moduli around 10–35 kPa (values are slightly higher for intact versus opened arteries). But TCFA specifically have not been examined, which needs to be done. The large applied stresses used to date in OCTE studies (particularly those used with acoustical radiation pressure or photo acoustic impulses) are much higher than 35 kPa [81–86]. Using strains (ultrasound amplitude differences) in the range of the weak TCFAs will increase differentiation of the small differences among TCFAs as well as the results being near linear. These TCFA moduli can be measured by applying a series of weights *simultaneously* assessing displacement (measured both optically and with calipers). This is a procedure we have previously described and review below [42–43].

#### 2. Compensating for the finite tissue response time

Again, the finite tissue strain response times need to be compensated for at high data acquisition rates. In other words, strain needs to remain relatively constant during the B-scan for an accurate measurement of the modulus. As stated, it takes approximately 20 msec to reach steady state once stress is applied. This is an issue at video rate as each frame is about 30 msec so the strain is continuously changing (Figure 2). As in Figure 3, we will overcome this problem by applying stress with constant frequency, amplitude-modulated ultrasound in alternate frames. The amplitude is changed between two values. The strain variability is reduced by performing elastography measurements on the 2^nd^ and 4^th^ frames while transitioning amplitudes on the 1st and 3rd frames.

#### 3. Dealing with SS-OCT specific issue

The approach needs to be tailored to SS-OCT currently used for intracoronary systems (most TD-OCT elastography approaches are not transferable to SS-OCT). With SS-OCT A- and B-scans are reconstructed and indirectly measured. So with SS-OCT, the variable strain from the tissue dynamic properties alters the autocorrelation function (unlike TD-OCT) as individual frequencies used to reconstruct it are altered unpredictably. The alternating frames, as in Figure 3, are also used to deal with this issue specific to SS-OCT.

#### 4. Maintaining a constant pre-load

Preload needs to be controlled as it alters the value of the measured modulus. This will be achieved by keeping the lower of the two-ultrasound amplitudes constant and above diastolic pressure.

#### 5. Controlling conditioning

Conditioning needs to be controlled as it alters the value of the measured modulus. A constant ultrasound carrier frequency will be used to achieve this.

#### 6. Area of applied stress

The area of applied stress needs to be kept smaller than the TCFA. This is to avoid compressing the edges that would alter modulus measurements.

#### 7. Sampling area

In our previous examination of fibroatheromas and fibrous plaque for OCTE, the area assessed is 50×50 pixels, with a 21×21 kernel window, and 500 μm by 50 μm imaging area. There is no reason to suspect that a different window size will be needed for TCFAs. The reason is that the amount of depression will be less (not more) because of the reduced stress.

## Discussion

OCTE has the potential of being a powerful diagnostic tool across a wide range of disorders from osteoarthritis to oncology. Here, to illustrate the importance of advancing the OCTE field, we have focused on using OCTE for the prevention of MI by identifying weak TCFAs. Many OCTE approaches have been examined to date and are reviewed elsewhere [47–57,59–74]. This paper illustrates current OCTE approaches fail to fully take into account most critically dynamic tissue properties and to a lesser extent static tissue properties. The most critical conclusion of the paper is that these limit current OCTE accuracy at video rates.

We are not going to examine these limitations for all the different OCTE approaches (just two). Instead we have discussed the five components that all OCTE techniques are a combination of. By doing this, the limitations imposed by dynamic and static tissue responses for video rate imaging are clear for all OCTE techniques. Many papers simply divide OCTE techniques by their excitation into static/quasi-static and dynamic, but this ignores these tissue responses that introduce error. So it should be clear from the work in this paper that the technically elegant dynamic excitation approaches become unreliable at high frame rates primarily because of the dynamic and static tissue properties. The more recent dynamic excitation approaches of phase-resolved acoustic radiation force optical coherence elastography method (ARF-OCE) and OCT alternating line elastography (OCTALE) are obvious examples [68]. The ARF-OCE method utilizes chirped acoustic radiation force to produce excitation along the sample’s axial direction, and it uses a phase-resolved OCT system to measure the vibration of the sample. Under usually 500-Hz square wave modulated ARF signal excitation, phase change maps of tissue mimicking phantoms are produced by the ARF-OCE method. While this technique has generated considerable attention, it has not been validated against standards. But more relevant to the paper, the dynamic tissue properties result in the modulus varying unpredictably at video rate. The second example is the relatively recent OCT alternating line elastography (OCTALE) technique. It employs the correlation between the overlapping point-spread functions (PSF) of adjacent A-scans, instead of comparing subsequent B-scans. The faulty assumption with this approach is that at video rate, the strain for a given stress will be the same for each A-scan making the overlap of the lateral PSF viable to use. But as outlined in the paper, the tissue strain response is complex and changing, making this an invalid assumption. In addition, the modulus (between A-scans) is altered by the changing preloads and afterloads. Because the dynamic tissue response can’t be (and aren’t) accounted for these popular dynamic excitation techniques, we strongly support the static/quasi-static based approaches for real time OCTE.

In this paper, we presented an approach that used CW excitation changing in alternate frame. It illustrates how to overcome challenges posed by dynamic tissue properties to allow in vivo real time application. System parameters were chosen based on current knowledge of tissue characteristics in the vascular system but can be adjusted to optimize for different organ systems. The system is designed to easily alter parameters for optimizing performance. Parameters that can be altered include amplitude gaps, carrier frequency, window size, beam size, pixel density, which frames are sampled, and the use of alternative speckle algorithms. For example, if a given tissue the response time (on preliminary testing) is longer than the frame rate, approaches to deal with this include increasing preload (increase the lower amplitude), increasing conditioning (carrier frequency), or using every third rather than every second frame. In another example, if the amplitude differences tested result in insufficient differentiation of tissue types (ex: different TCFAs), the amplitude difference can simply be increase (with likely some modification of the window size). The opposite would be true if decorrelation is an issue for a pathology of interest [90,91]. The amplitude difference and window size would be decreased (with higher sampling).

In addition to dynamic properties, static tissue properties also needed to be accounted for. Here, to maintain linearity, the applied pressures need to be near the tissue moduli and the beam size appropriately chosen. As discussed, as part of incorporating static tissue properties, we need to establish the range of tissue moduli to optimize the applied pressures (ultrasound amplitude differences). Prior OCTE studies on atherosclerotic arteries, including by our group, used stresses much higher (>100 kPa) than the moduli of lipid plaque (<50 kPa based on the limited data in the literature). But no group has studied TCFA moduli specifically, just plaque in general. By optimizing the system around the TCFA moduli, relevant plaque differentiation can be more sharply defined and linearity maintained. A common procedure for determining these moduli, we have published previously, involves applying a series of weights to the center of the plaques with a tripod [91,92]. The tripod avoids uncertainties from a suspension bar or from free weight movement. The diameter of the weights is the same as the ultrasound beam (2 mm), which will be smaller than the plaque width, eliminating edge effects (distorting the modulus). The objective is to find at least 5 weights that encompass the modulus range of TCFAs. In the past we have measured the compressions from these weights optically (OCT) and with calipers [93,94]. This gives stress (weight) and strain (compression) profiles for the plaque moduli calculation. With this knowledge, it is repeated with ultrasound and the ultrasound amplitudes to achieve pressures in this range determined.

We have also noted how the tissue needs to be sampled is dictated to a large degree by the static tissue properties. Sampling, of course, can only be over a limited region of the plaque but it needs to be representative. From a practical standpoint, ‘representative’ clinically means able to differentiate the plaques of interest and doesn’t necessarily imply being technically precise or an idealized measurement (i.e. needs to be repeatable). But in general, the tissue has to behave as if it is uniform, incompressible, and an isotropic linear elastic material for repeatable measurements. We have already discussed for the plaque of interest this is a reasonable assumption. Several critical factors hold for these plaques allowing linearity to be an excellent assumption (holds within 2% of the actual modulus). These are 1) the lipid core strain is higher than the surrounding tissue, 2) the core is larger laterally than axially, and 3) compressions are less than 5% [81–86]. The first two points are the nature of TCFAs and fibro atheroma while the last point is achieved by restricting the applied stress to around the TCFA moduli.

We are also concerned about the size of the sampling area. Too small and it doesn’t give representative sampling and too large edge effects occur. In general, previous data has shown small heterogeneous lipid cores the size of the TCFAs behave as if they are relatively homogeneous [30,40,84,85,92,93]. Based on this, the data supports regional imaging of a 500 by 500 μm area under the cap center to be effective. This is chosen for several reasons. First it is smaller than the ultrasound beam (2 mm) so there are no edge effects which prevents compression of the periphery of the cap (with ultrasound pressure) that could give an artificially high modulus. Second, compression from above (and central) is consistent with models of in vivo diastolic stress that leads to TCFA rupture.

### Limitations

This paper has focused on tissue characteristics (dynamic and static) that need to be accounted for to achieve accurate measurements of moduli at video rate. The ultrasound-OCT catheter, needed for in vivo imaging, has not been the focus and likely initial studies will be performed in vitro with open arteries. There are two reasons for this. One reason is that optimizing performance in vitro will be needed before the best design of the catheter can be established. The second reason is several potential designs exist and this would be the source of a paper itself. The major issue is how the ultrasound is applied, as the catheter is otherwise the same. The OCT catheter optics are less than 400 μm so incorporating ultrasound for transmitting will not significantly effect catheter diameter. The ultrasound needs to be a beam at 2 mm in diameter or less so ring transducers used with optoacoustical imaging are not an option [94]. It is likely a phase array deliver system will be used similar to some IVUS designs as this will allow a focused application of the ultrasound. Since the focus of this paper is overcoming the limitations, at video rate, of dynamic and static issue characteristics, the issue of catheter design will not be discussed in any more detail.

## Conclusion

Current OCTE approaches can’t be scaled up to the needs for in vivo imaging, particularly intravascular imaging where it likely will have a major impact. Both dynamic and static tissue properties are not considered in their design, resulting in inaccuracies at video rate. Factors preventing this up-scaling include finite tissue response time, varying preloads, and varying conditioning. In this paper we propose an OCTE approach for in vivo real time imaging that addresses these challenges and is viable for intravascular imaging. While it is relevant to other pathologies, it offers the opportunity of being the technology that can differentiate stable from unstable plaque to prevent myocardial infarction.

## Figures and Tables

**Table 1:** Computation time for two images of 50*50 pixels, at variant pixel rate and window size.

Pixel rate	Every pixel	Every other pixel	Every third pixel	Every fifth pixel
Window size				
21 pixels	16.3 sec	4.3 sec	1.9 sec	0.9 sec
31 pixels	15.4 sec	3.7 sec	1.7 sec	0.8 sec

## References

[R1] 1http://www.cdc.gov/nchs/deaths.htm

[2] Falk E (1983). Plaque rupture with severe pre-existing stenosis precipitating coronary thrombosis. Characteristics of coronary atherosclerotic plaques underlying fatal occlusive thrombi. Br Heart J.

[3] Richardson PD, Davies MJ, Born GVR (1989). Influence Of Plaque Configuration And Stress Distribution On Fissuring Of Coronary Atherosclerotic Plaques.

[4] Virmani R, Kolodgie FD, Burke AP, Farb A, Schwartz SM (2000). Lessons from sudden coronary death: A comprehensive morphological classification scheme for atherosclerotic lesions. Arterioscler Thromb Vasc Biol.

[5] Virmani R, Burke AP, Farb A, Kolodgie FD (2002). Pathology of the unstable plaque. Prog Cardiovasc Dis.

[6] Cheruvu PK, Finn AV, Gardner C, Caplan J, Goldstein J (2009). Frequency and distribution of thin-cap fibroatheroma and ruptured plaques in human coronary arteries: a pathologic study. J Am Coll Cardiol.

[7] Sluimer JC, Kolodgie FD, Bijnens AP, Maxfield K, Pacheco E (2009). Thin-walled microvessels in human coronary atherosclertic plaques show incomplete endothelial junctions relevance of compromised structural integrity for intraplaque microvascular leakage. J Am Coll Cardiol.

[8] Finn AV, Nakano M, Narula J, Kolodgie FD, Virmani R (2010). Concept of vulnerable/unstable plaque. Arterioscler Thromb Vasc Biol.

[9] Stone GW, Maehara A, Lansky AJ, de Bruyne B, Cristea E (2011). A prospective natural-history study of coronary atherosclerosis. N Engl J Med.

[10] Fleg JL, Stone GW, Fayad ZA, Granada JF, Hatsukami TS (2012). Detection of high-risk atherosclerotic plaque: report of the NHLBI Working Group on current status and future directions. JACC Cardiovasc Imaging.

[11] Narula J, Nakano M, Virmani R, Kolodgie FD, Petersen R (2013). Histopathologic characteristics of atherosclerotic coronary disease and implications of the findings for the invasive and noninvasive detection of vulnerable plaques. J Am Coll Cardiol.

[12] Brodie B, Pokharel Y, Garg A, Kissling G, Hansen C (2012). Predictors of early, late, and very late stent thrombosis after primary percutaneous coronary intervention with bare-metal and drug-eluting stents for ST-segment elevation myocardial infarction. JACC Cardiovasc Interv.

[13] Ambrose JA, Tannenbaum MA, Alexopoulos D, Hjemdahl-Monsen CE, Leavy J (1988). Angiographic progression of coronary artery disease and the development of myocardial infarction. J Am Coll Cardiol.

[14] Little WC, Constantinescu M, Applegate RJ, Kutcher MA, Burrows MT (1988). Can coronary angiography predict the site of a subsequent myocardial infarction in patients with mild-to-moderate coronary artery disease?. Circulation.

[15] Rioufol G, Finet G, Ginon I, André-Fouët X, Rossi R (2002). Multiple Atherosclerotic Plaque Rupture in Acute Coronary Syndrome: A Three Vessel Intravascular Ultrasound Study. Circulation.

[16] Goldstein JA, Demetriou D, Grines CL, Pica M, Shoukfeh M (2000). Multiple complex coronary plaques in patients with acute myocardial infarction. N Engl J Med.

[17] Lee RT, Grodzinsky AJ, Frank EH, Kamm RD, Schoen FJ (1991). Structure-dependent dynamic mechanical behavior of fibrous caps from human atherosclerotic plaques. Circulation.

[18] Loree HM, Kamm RD, Stringfellow RG, Lee RT (1992). Effects of fibrous cap thickness on peak circumferential stress in model atherosclerotic vessels. Circulation Research.

[19] Brezinski ME, Harjai K (2014). Longitudinal Necrotic Shafts Near TCFAs - A Potential Novel Mechanism for Plaque Rupture to Trigger ACS?. International Journal of Cardiology.

[20] Brezinski ME, Tearney GJ, Bouma BE, Izatt JA, Hee MR (1996). Optical coherence tomography for optical biopsy-Properties and demonstration of vascular pathology. Circulation.

[21] Huang D, Swanson EA, Lin CP, Schuman JS, Stinson WG (1991). Optical Coherence Tomography. Science.

[22] Brezinski ME (2006). Optical Coherence Tomography: Principle and Practice.

[23] Fujimoto JG, Boppart SA, Tearney GJ, Bouma BE, Pitris C (1999). High resolution in vivo intra-arterial imaging with optical coherence tomography. Heart.

[24] Yabushita H, Bourna BE, Houser SL, Aretz T, Jang IK (2002). Characterization of human atherosclerosis by optical coherence tomography. Circulation.

[25] Manfrini O, Mont E, Leone O, Arbustini E, Eusebi V (2006). Sources of error and interpretation of plaque morphology by the optical coherence tomography. Am J Cardiol.

[26] Kume T, Akasaka T, Kawamoto T, Okura H, Watanabe N (2006). Measurement of the thickness of the fibrous cap by optical coherence tomography. Am Heart J.

[27] Jang IK, Tearney GJ, MacNeill B, Takano M, Moselewski M (2005). In vivo characterization of coronary atherosclerotic plaque by use of optical coherence tomography. Circulation.

[28] Kubo T, Imanishi T, Takarada S, Kuroi A, Ueno S (2007). Assessment of culprit lesion morphology in acute myocardial infarction-Ability of optical coherence tomography compared with intravascular ultrasound and coronary angioscopy. J Am Coll Cardiol.

[29] Brezinski ME (2011). Current Capabilities and Challenges for Optical Coherence Tomography as a High-Impact Cardiovascular Imaging Modality. Circulation.

[30] Brezinski ME (2006). Optical coherence tomography for identifying unstable coronary plaque. Int J Cardiol.

[31] Cheng GC, Loree HM, Kamm RD, Fishbein MC, Lee RT (1993). Distribution of circumferential stress in ruptured and stable atherosclerotic lesions. A structural analysis with histopathological correlation. Circulation.

[32] Richardson PD (2002). Biomechanics of Plaque Rupture: Progress, Problems, and New Frontiers. Annals of Biomedical Engineering.

[33] Liang X, Xenos M, Alemu Y, Rambhia SH, Lavi I (2013). Biomechanical factors in coronary vulnerable plaque risk of rupture: intravascular ultrasound-based patient-specific fluid-structure interaction studies. Coron Artery Dis.

[34] De Korte CL, van derSteen A, Céspedes E, Pasterkamp G (1998). Intravascular ultrasound elastography in human arteries: initial experience in vitro. Ultrasound Med Biol.

[35] De Korte CL, Céspedes EI, van der Steen AF, Lancée CT (1998). Intravascular elasticity imaging using ultrasound: feasibility studies in phantoms. Ultrasound Med Biol.

[36] Patwari P, Weissman NJ, Boppart SA, Jesser C, Stamper D (2000). Assessment of coronary plaque with optical coherence tomography and high-frequency ultrasound. Am J Cardiol.

[37] Schaar JA, De Korte CL, Mastik F, Strijder C, Pasterkamp G (2003). Characterizing vulnerable plaque features with intravascular elastography. Circulation.

[38] Schaar JA, Regar E, Mastik F, McFadden EP, Saia F (2004). Incidence of high-strain patterns in human coronary arteries: assessment with three-dimensional intravascular palpography and correlation with clinical presentation. Circulation.

[39] Schaar JA, van der Steen AF, Mastik F, Baldewsing RA, Serruys PW (2006). Intravascular palpography for vulnerable plaque assessment. J Am Coll Cardiol.

[40] Ophir J, Céspedes I, Ponnekanti H, Yazdi Y, Li X (1991). Elastography: a quantitative method for imaging the elasticity of biological tissues. Ultrason Imaging.

[41] Li YF, Snedeker JG (2011). Elastography: modality-specific approaches, clinical applications, and research horizons. Skeletal Radiol.

[42] Brezinski ME, Tearney GJ, Bouma BE, Izatt JA, Hee MR (1996). Optical coherence tomography for optical biopsy-Properties and demonstration of vascular pathology. Circulation.

[43] Huang D, Swanson EA, Lin CP, Schuman JS, Stinson WG (1991). Optical Coherence Tomography. Science.

[44] Brezinski ME (2006). Optical Coherence Tomography: Principle and Practice.

[45] Brezinski ME, Harjai K (2014). Current OCT Approaches Do Not Reliably Identify TCFAs. Journal of Clinical and Experimental Cardiology.

[46] Schmitt M, Xiang SH, Yung KM (1999). Speckle in optical coherence tomography. J Biomed Opt.

[47] Rogowska J, Patel N, Plummer S, Brezinski ME (2006). Quantitative optical coherence tomographic elastography: method for assessing arterial mechanical properties. Br J Radiol.

[48] Liang X, Orescanin M, Toohey KS, Insana MF, Boppart SA (2009). Acoustomotive optical coherence elastography for measuring material mechanical properties. Optics Letters.

[49] Oldenburg AL, Boppart SA (2010). Resonant acoustic spectroscopy of soft tissues using embedded magnetomotive nanotransducers and optical coherence tomography. Phys Med Biol.

[50] Crecea V, Oldenburg AL, Liang X, Ralston TS, Boppart SA (2009). Magnetomotive nanoparticle transducers for optical rheology of viscoelastic materials. Opt Express.

[51] Wang S, Li JS, Manapuram RK, Menodiado FM, Ingram DR (2012). Noncontact measurement of elasticity for the detection of soft-tissue tumors using phase-sensitive optical coherence tomography combined with a focused air-puff system. Opt Lett.

[52] Cheng Y, Li R, Li SN, Dunsby C, Eckersley RJ, Elson DS (2012). Shear Wave Elasticity Imaging Based On Acoustic Radiation Force And Optical Detection. Ultrasound Med Biol.

[53] Huang C, Liu B, Brezinski ME (2008). Ultrasound-enhanced optical coherence tomography: Improved penetration and resolution. Journal of The Optical Society Of America A-Optics Image Science And Vision.

[54] Kennedy BF, Koh SH, McLaughlin RA, Kennedy KM, Munro PR (2012). Strain estimation in phase-sensitive optical coherence elastography. Biomed Opt Express.

[55] Adie SG, Liang X, Kennedy BF, John R, Sampson DD (2010). Spectroscopic optical coherence elastography. Opt Express.

[56] Liang X, Adie SG, John R, Boppart SA (2010). Dynamic spectral-domain optical coherence elastography for tissue characterization. Opt Express.

[57] Blatter C, Grajciar B, Zou P, Wieser W, Verhoef AJ (2012). Intrasweep phase-sensitive optical coherence tomography for noncontact optical photoacoustic imaging. Opt Lett.

[58] Wang RKK, Kirkpatrick S, Hinds M (2007). Phase-sensitive optical coherence elastography for mapping tissue microstrains in real time. Applied Physics Letters.

[59] Wang RKK, Ma ZH, Kirkpatrick SJ (2006). Tissue Doppler optical coherence elastography for real time strain rate and strain mapping of soft tissue. Applied Physics Letters.

[60] Li CH, Guan GY, Reif R, Huang ZH, Wang RK (2012). Determining elastic properties of skin by measuring surface waves from an impulse mechanical stimulus using phase-sensitive optical coherence tomography. J R Soc Interface.

[61] Li CH, Guan GY, Cheng X, Huang ZH, Wang RK (2012). Quantitative elastography provided by surface acoustic waves measured by phase-sensitive optical coherence tomography. Opt Lett.

[62] Doherty JR, Dumont DM, Trahey GE, Palmeri ML (2013). Acoustic radiation force impulse imaging of vulnerable plaques: a finite element method parametric analysis. J Biomech.

[63] Qi W, Chen R, Chou L, Liu G, Zhang J (2012). Phase-resolved acoustic radiation force optical coherence elastography. J Biomed Opt.

[64] Kennedy BF, Wojtkowski M, Szkulmowski M, Kennedy KM, Karnowski V (2012). Improved measurement of vibration amplitude in dynamic optical coherence elastography. Biomed Opt Express.

[65] Liang X, Boppart SA (2010). Biomechanical Properties of In Vivo Human Skin From Dynamic Optical Coherence Elastography. IEEE Trans Biomed Eng.

[66] Dumont DM, Doherty JR, Trahey GE (2011). Noninvasive assessment of wall-shear rate and vascular elasticity using combined ARFI/SWEI/spectral Doppler imaging system. Ultrason Imaging.

[67] Khalil S, Bouma BE, Mofrad BEK (2006). A combined FEM/genetic algorithm for vascular soft tissue elasticity estimation. Cardiovasc Eng.

[68] Karimi R, Zhu T, Bouma BE, Mofrad MRK (2008). Estimation of Nonlinear Mechanical Properties of Vascular Tissues via Elastography. Cardiovasc Eng.

[69] Bonnet M, Constantinescu A (2005). Inverse problems in elasticity. Inverse Prob.

[70] Krouskop TA, Wheeler TM, Kallel F, Garra BS, Hall T (1998). Elastic moduli of breast and prostate tissues under compression. Ultrason Imaging.

[71] Erkamp R, Wiggins P, Skovoroda A, Emelianov S, Donnell MO (1998). Measuring the elastic modulus of small tissue samples. Ultrason Imaging.

[72] Liu GJ, Chou L, Jia WC, Qi WJ, Choi B (2011). Intensity-based modified Doppler variance algorithm: application to phase instable and phase stable optical coherence tomography systems. Opt Express.

[73] Manapuram RK, Aglyamov S, Menodiado FM, Mashiatulla M, Wang S (2012). Estimation of shear wave velocity in gelatin phantoms utilizing PhSSSOCT. Laser Physics.

[74] Manapuram RK, Aglyamov SR, Monediado FM, Mashiatulla M, Li JS (2012). In vivo estimation of elastic wave parameters using phase-stabilized swept source optical coherence elastography. J Biomed Opt.

[75] Rogowska J, Patel NA, Fujimoto JG, Brezinski ME (2004). Optical coherence tomographic elastography technique for measuring deformation and strain of atherosclerotic tissues. Heart.

[76] Duncan D, Kirkpatrick SJ (2001). Processing algorithms for tracking speckle shifts in optical elastography of biological tissues. J Biomed Opt.

[77] Kirkpatrick SJ, Wang RK, Duncan DD (2006). OCT-based elastography for large and small deformations. Opt Express.

[78] Barrett SR, Sutcliffe MP, Howarth S, Li ZY, Gillard (2009). Experimental measurement of the mechanical properties of carotid atherothrombotic plaque fibrous cap. J Biomech.

[79] Srivastava, Verma Y, Rao KD, Gupta PK (2011). Determination of Elastic Properties of Resected Human Breast Tissue Samples Using Optical Coherence Tomographic Elastography. Strain.

[80] Samani A, Bishop J, Luginbuhl C, Plewes DB (2003). Measuring the elastic modulus of ex vivo small tissue samples. Phys Med Biol.

[81] Loree HM, Tobias BJ, Gibson LJ, Kamm RD, Small DM (1994). Mechanical properties of model atherosclerotic lesion lipid pools. Arterioscler Thromb.

[82] Lerner R, Huang S, Parker K (1990). Sonoelasticity images derived from ultrasound signals in mechanically vibrated tissues. Ultrasound Med Biol.

[83] Parker K, Huang S, Musulin R, Lerner R (1990). Tissue response to mechanical vibrations for sonoelasticity imaging. Ultrasound Med Biol.

[84] Adie SG, Kennedy BF, Armstrong JJ, Alexandrov SA, Sampson DD (2009). Audio frequency in vivo optical coherence elastography. Phys Med Biol.

[85] Lamouche SF, Kennedy BF, Kennedy KM, Bisaillon CE, Curatolo A (2012). Review of tissue simulating phantoms with controllable optical, mechanical and structural properties for use in optical coherence tomography. Biomed Opt Express.

[86] Van Soest G, Bouchard RR, Mastik F, De Jong N, Van der Steen AFW (2007). Robust intravascular optical coherence elastography driven by acoustic radiation pressure. OSA Biomedical Optics.

[87] Williamson SD, Lam Y, Younis HF, Huang H, Patel S (2003). On the Sensitivity of Wall Stresses in Diseased Arteries to Variable Material Properties. J Biomech Eng.

[88] Khalil AS, Chan RC, Chau AH, Bouma BE, Mofrad MRK (2005). Tissue elasticity estimation with optical coherence elastography: Toward mechanical characterization of In vivo soft tissue. Ann Biomed Eng.

[89] Liu B, Azimi E, Brezinski ME (2011). True logarithmic amplification of frequency clock in SS-OCT for calibration. Biomed Opt Express.

[90] Steckenrider JS, Wagner JW (1995). Computed Speckle Decorrelation (CSD) For The Study Of Fatigue Damage. Optics And Lasers In Engineering.

[91] Nadkarni SK, Bouma BE, Helg T, Chan R, Halpern E (2005). Characterization of atherosclerotic plaques by laser speckle imaging. Circulation.

[92] Vakoc BJ, Tearney GJ, Bouma BE (2009). Statistical Properties of Phase-Decorrelation in Phase-Resolved Doppler Optical Coherence Tomography. IEEE Trans Med Imaging.

[93] Azimi E, Liu B, Brezinski ME (2010). Real-time and high performance calibration method for high-speed swept-source optical coherence tomography. J Biomed Opt.

[94] Brezinski ME, Harjai K (2014). Current OCT Approaches Do Not Reliably Identify TCFAs.

